# Safe- and sustainable-by-design: The case of Smart Nanomaterials. A perspective based on a European workshop

**DOI:** 10.1016/j.yrtph.2021.105093

**Published:** 2022-02

**Authors:** Agnieszka Mech, Stefania Gottardo, Valeria Amenta, Alessia Amodio, Susanne Belz, Søren Bøwadt, Jana Drbohlavová, Lucian Farcal, Paula Jantunen, Aleksandra Małyska, Kirsten Rasmussen, Juan Riego Sintes, Hubert Rauscher

**Affiliations:** a European Commission, Joint Research Centre (JRC), Ispra, Italy; b European Commission, Directorate-General Research and Innovation (DG RTD), Brussels, Belgium

**Keywords:** Smart nanomaterial, Safe-by-Design, Safe- and Sustainable-by-Design, Regulatory requirements, European Union policy

## Abstract

The European Commission's Green Deal is a major policy initiative aiming to achieve a climate-neutral, zero-pollution, sustainable, circular and inclusive economy, driving both the New Industrial Strategy for Europe and the Chemicals Strategy for Sustainability. Innovative materials can help to reach these policy goals, but they need to be safe and sustainable themselves. Thus, one aim is to shift the development of chemicals to Safe- and Sustainable-by-Design, and define a new systems approach and criteria for sustainability to achieve this. An online workshop was organised in September 2020 by the Joint Research Centre and the Directorate-General Research and Innovation of the European Commission, with participants from academia, non-governmental organisations, industry and regulatory bodies. The aims were to introduce the concept of Safe- and Sustainable-by-Design, to identify industrial and regulatory challenges in achieving safer and more sustainable Smart Nanomaterials as an example of innovative materials, and to deliver recommendations for directions and actions necessary to meet these challenges. The following needs were identified: (i) an agreed terminology, (ii) a common understanding of the principles of Safe- and Sustainable-by-Design, iii) criteria, assessment tools and incentives to achieve a transition from Safe-by-Design to Safe- and Sustainable-by-Design, and (iv) preparedness of regulators and legislation for innovative chemicals/nanomaterials. This paper presents the authors' view on the state of the art as well as the needs for future activities, based on discussions at the workshop and further considerations. The case of Smart Nanomaterials is used to illustrate the Safe- and Sustainable-by-Design concept and challenges for its implementation. Most of the considerations can be extended to other advanced materials and to chemicals and products in general.

## Highlights

• Safe- and Sustainable-by-Design chemicals contribute to the transformation of policy ambitions into a circular economy.
• Smart nanomaterials are a case study of the challenges of Safe- and Sustainable-by-Design.
• The development of criteria to guide the design of chemicals facilitates achieving sustainability.
• Broad SSbD expert consultation is needed for consensus on principles and implementation.
• Agreed terminology and assessment tools for Smart Nanomaterials help to achieve the EU green policy goals.

## Abbreviations

CPR: Cosmetic Products Regulation
(DG) RTD: (Directorate-General) Research and Innovation
EC: European Commission
ECHA: European Chemicals Agency
EC NM Definition: European Commission Recommendation on a definition of the term nanomaterial
EFSA: European Food Safety Authority
EU: European Union
FAIR: findable, accessible, interoperable and reusable
ISO: International Organization for Standardization
JRC: Joint Research Centre
NM: nanomaterial
LCA: Life Cycle Assessment
OECD: Organisation for Economic Co-operation and Development
R&D: research and development
REACH: legislation on Registration, Evaluation, Authorisation and Restriction of Chemicals
RP: Regulatory Preparedness
RRI: Responsible Research and Innovation
SbD: Safe-by-Design
SCCS: Scientific Committee on Consumer Safety
SSbD: Safe- and Sustainable-by-Design
SIA: Safe Innovation Approach
Smart NMs: Smart nanomaterials
TRL: Technology Readiness Level
UN: United Nations

## 1 Introduction

The European Commission's (EC) European Green Deal (European Commission, 2019) published in 2019 is a major policy initiative which aims to achieve a climate-neutral, zero-pollution, sustainable, circular and inclusive economy. It is driving a New Industrial Strategy for Europe (European Commission, 2020a) that promotes responsible design and development of materials and products by maximising their safety, lifetime and potential for reuse and recycling while minimising adverse effects on human health and the environment. The Green Deal also guides the European Union's (EU) Chemicals Strategy for Sustainability Towards a Toxic-free Environment (European Commission, 2020b), which aims to simplify and strengthen the regulatory framework for chemicals to further increase the level of protection of human health and the environment while boosting the competitiveness of the EU chemicals industry. It specifically states that the capacity of manufacturing new chemicals that are “inherently safe and sustainable from production to end of life” will play a crucial role in the green and digital transitions, and envisages the shift towards a “safe and sustainable-by-design approach for chemicals” (Box 1), to be ensured by adequate regulation. Table 1 provides a list of key Communications by the EC on these policy initiatives.

Box 1 **Safe-by-Design (OECD, 2020) (OECD, 2020) The SbD (Safe-by-Design, Safer-by-Design, or Safety-by-Design) concept refers to identifying the risks and uncertainties concerning humans and the environment at an early phase of the innovation process so as to minimize uncertainties, potential hazard(s) and/or exposure. The SbD approach addresses the safety of the material/product and associated processes through the whole life cycle: from the Research and Development (R&D) phase to production, use, recycling and disposal.**

*For SbD in nanotechnology, three pillars of design can be specified:*

I. *Safe(r) material/product: minimising, in the R&D phase, possible hazardous properties of the nanomaterial or nano-enabled product while maintaining function;*
II. *Safe(r) production: ensuring industrial safety during the production of nanomaterials and nano-enabled products, more specifically occupational, environmental and process safety aspects; and*
III. *Safe(r) use and end-of-life: minimising exposure and associated adverse effects through the entire use life, recycling and disposal of the nanomaterial or nano-enabled product. This can also support circular economy.*

**Safe and Sustainable-by-Design(European Commission 2020)** (European Commission, 2020b) *At this stage, safe and sustainable-by-design can be defined as a pre-market approach to chemicals that focuses on providing a function (or service), while avoiding volumes and chemical properties that may be harmful to human health or the environment, in particular groups of chemicals likely to be (eco) toxic, persistent, bio-accumulative or mobile. Overall sustainability should be ensured by minimising the environmental footprint of chemicals in particular on climate change, resource use, ecosystems and biodiversity from a lifecycle perspective*.

Alt-text: Box 1

**Table 1:** European Commission key Communications on policy initiatives related to the Green Deal and referred to in the text.

Document title	Comment	Document number
**A European Green Deal**. Communication from the Commission to the European Parliament, the European Council, the Council, the European Economic and Social Committee and the Committee of the Regions	*A set of policy initiatives by the European Commission with the overarching aim of making Europe climate neutral in 2050.*	COM(2019) 640 final
**EU's Chemicals Strategy for Sustainability Towards a Toxic-Free Environment**. Communication from the Commission to the European Parliament, the European Council, the Council, the European Economic and Social Committee and the Committee of the Regions	*As part of the European Green Deal's zero-pollution vision, the strategy aims to better protect human health, strengthen business competitiveness, and support a toxic-free environment.*	COM(2020) 667 final
**A New Industrial Strategy for Europe**. Communication from the Commission to the European Parliament, the European Council, the Council, the European Economic and Social Committee and the Committee of the Regions.	*Lays the foundations for an industrial strategy that would support the twin transition to a green and digital economy to make EU industry more competitive globally, and enhance Europe's open strategic autonomy.*	COM(2020) 102 final
**A new Circular Economy Action Plan for a cleaner and more competitive Europe**. Communication from the Commission to the European Parliament, the Council, the European Economic and Social Committee and the Committee of the Regions	*One of the main building blocks of the EU Green Deal, it announces initiatives along the entire life cycle of products towards the transition to a circular economy.*	COM(2020) 98 final

When the legislation is fit for purpose, the safety of a process, material or product can in principle be demonstrated by fulfilling specific regulatory requirements. One example are the safety related requirements in the EU Regulation on Registration, Evaluation, Authorisation and Restriction of Chemicals (REACH) (European Parliament and Council, 2006). Ensuring human health and environmental protection by addressing chemical safety in legislation is also a step towards sustainability. In 1987, the United Nations (UN) defined sustainable development (UN World Commission on Environment and Development, 1987) as “*meeting the needs of the present without compromising the ability of future generations to meet their own needs*”, and it is currently understood to cover social, economic and environmental aspects. In addition, the UN has also agreed on, and continuously updates, the Sustainable Development Goals (United Nations, 2020).^.^ Agenda 21 (United Nations, 1992) from 1992 was the first global action plan setting goals and a timeframe for achieving them (by 2000), and it has been followed up with additional goals (United Nations, 2002, 2015). Reflecting an evolution in thinking, the current goals are presented individually but are seen as being interconnected, requiring a new systems approach to reach them. At present, ways to reach individual UN Sustainable Development Goals mentioned in the Agenda 21 are well reflected in EU legislation; references to these goals and to sustainability are often given at high level. In REACH, for example, they are stated in the preamble among the international treaties the regulation addresses. The legal provisions include requirements that ensure safety, but there are no specific requirements to demonstrate that a substance is manufactured or can be used in a sustainable way.

For the systems approach and as an important contribution to sustainable development, chemical processes and products must be adapted to fit into a circular economy (European Commission, 2020c), which is a concept aimed at minimising waste, reusing and recycling products, saving resources and preserving the environment, i.e. striving for sustainability. Obviously, this includes identifying suitable starting materials and modifying them to the desired grade, structure, and function for a specific application (Kümmerer et al., 2020).

The Safe-by-Design (SbD)^2^ concept applied to innovation in nanotechnology aims at the development of functional and safe nanomaterials and nano-enabled products (Kraegeloh et al., 2018). The approach optimises the use of resources and expedites the development of new nanomaterials and nano-enabled products that are safe by design (OECD, 2020). In a similarly systematic approach, the emerging concept of Sustainable-by-Design aims to address the sustainability aspects of materials and products under development (Babbitt and Moore, 2018). The recently advocated Safe- and Sustainable-by-Design (SSbD) approach combines aspects of the two concepts (Gottardo et al., 2021).

It is important that the proactive implementation of these concepts by innovators is accompanied by Regulatory Preparedness (RP) (Jantunen et al., 2018a), which helps regulators to keep up with innovation and to prepare appropriate legislation and other regulatory tools in good time for their arrival to the market. The EU Horizon 2020 project NanoReg2 (Soeteman-Hernandez et al., 2019) was built on the projects NANoREG (NANoREG, 2021) and ProSafe (ProSafe, 2021) and combined the SbD and RP concepts into a Safe Innovation Approach (SIA), which was further developed for nanomaterials and nano-enabled products by the Organisation for Economic Co-operation and Development (OECD, 2020).

Nanomaterials (NM) are currently among the most innovative materials, and efforts to engineer nanomaterials with use-case tailored properties have resulted in complex structures. It has been suggested that nanotechnology will mature by moving through six increasingly sophisticated, overlapping generations, also reflecting a convergence of different disciplines of science and engineering (European Chemicals Agency, 2019a). Smart nanomaterials (Smart NMs), also known as stimuli-responsive, multifunctional or active nanomaterials (Gottardo et al., 2021; Municoy et al., 2020; Kim et al., 2018; Sims et al., 2017), are a specific type of innovative materials that probably represents the currently most advanced nanomaterials, and they are often multicomponent in nature. Smart NMs are designed to respond to one or more selected external biotic or abiotic stimuli, such as changes in temperature, pH, humidity, ionic strength, light or the activity of enzymes, by changing specific properties and key functions (Gottardo et al., 2021). The complex and dynamic nature of Smart NMs has raised questions regarding their safety and sustainability and the ability of current regulatory frameworks to address them (Gottardo et al., 2021).

Applications of Smart NMs in food, packaging and cosmetics have already been commercialised, and many more are at the research and development (R&D) stage as discussed in a recent review (Gottardo et al., 2021). In the cosmetics sector, one use of Smart NMs is targeted delivery of ingredients to skin. The most developed systems are based on stimuli-responsive nanocapsules which protect active ingredients from degradation until the target site is reached and release the ingredients in response to the external stimuli, as developed for instance in the research project PeptiCaps (PeptiCaps, 2018).

Nano-enabled agrochemicals are another example of application of Smart NMs. They may play an important role in developing more sustainable ways of producing food, presenting new opportunities for saving energy and other resources and reducing environmental impacts from agriculture. Nanomaterials for agricultural use can be made kinetically stable and be combined with new, designed (or “smart”) reactivity, which offers new ways to manage the timing of agrochemical application through stimuli-based release of the active ingredients. Nano-sized fertilisers and plant protection products can more easily enter plants to deliver nutrients and pesticides. The design of such nanomaterials can reduce resource use while improving yields, crop health and nutrition: the nutrients are made available when and where the crop needs them (Hofmann et al., 2020) with the help of e.g. nanosized sensors (Martins et al., 2020), (Spielman-Sun et al., 2020), (Zhang et al., 2020), (Wu et al., 2018), (Grillo et al., 2021). The technology development level of such applications is currently at laboratory and greenhouse testing.

The issues linked to the SSbD approach were explored at an online workshop on 9–10 September 2020, entitled “*Safe and Sustainable Smart Nanomaterials*” (European Commission Workshop, 2020), jointly organised by the European Commission's Joint Research Centre (JRC) and the Directorate-General (DG) for Research and Innovation (RTD). It had the dual purpose of a) introducing, for the first time, stakeholders outside of the EC to the concept of SSbD for chemicals and products and discussing the needs to further develop and implement SSbD, and b) discussing, as a case study, legislative and research needs as well as challenges for both industry and regulators in obtaining safe and sustainable Smart NMs. The workshop concentrated on the developing field of Smart NMs applied in agriculture, food, food packaging and cosmetics; other areas, such as biomedicine or electronics, were outside the scope of the workshop. The discussion was steered around the identification of possible directions and actions necessary to meet the identified challenges in the context of the Chemicals Strategy for Sustainability. Economic and social aspects of sustainability were not in the scope of the workshop.

The event was attended by more than 90 participants representing European legislators, industry, researchers and non-governmental organisations (NGOs) as well as speakers from the USA. It included breakout groups and on-line polls and was divided into four parts:

• “*Designing Smart Nanomaterials*” dedicated to the development of Smart Nanomaterials in selected industrial fields and to safety and sustainability issues considered during the development phase.
• “*From safe-by-design to safe-and-sustainable-by-design*” illustrating and discussing challenges specifically related to safety and sustainability of Smart NMs.
• “*Regulatory Preparedness*” addressing the legislative context of Smart NMs and some initiatives within specific areas of legislation.
• “*How to shift towards a more sustainable path? Implications of the Chemical Strategy for Sustainability*” dedicated to the priority areas of Sustainable-by-Design in view of Horizon Europe, the EU's 2021–2027 framework programme for research and innovation.

This paper presents the context of shifting from Safe-by-Design to Safe- and Sustainable-by-Design and the case study of Smart NMs, which illustrates the possible challenges associated with this shift. The main conclusion of the discussions at the workshop are described, as well as further considerations by the authors on the most urgent issues to be addressed to obtain safe and sustainable Smart NMs.

## 2 Shifting from safe-by-design to safe- and sustainable-by-design

Chemical safety has been legally addressed at EU level since 1967 with the agreement of the Dangerous Substances Directive (Council of the European Economic Community, 1967). This legislation has evolved and expanded significantly over time, and the directions of its evolution have been supported also by a deeper understanding of effects of chemicals on both human health and the environment. Among other things, this has led to legislation that limits the exposure of man and the environment to hazardous chemicals, addressing the working environment and environmental compartments (soil, water and air). REACH, which is part of the recast of the Dangerous Substances Directive (Council of the European Economic Community, 1967), is based on the principle that it is the responsibility of manufacturers, importers and downstream users to ensure that the substances that are placed on the market can be manufactured and used by consumers and (professional) workers in a way that does not adversely affect human health or the environment. To this end, companies are required to submit data and perform risk assessments for each registered substance and their identified uses. Guidance and tools have been developed to facilitate the implementation of REACH safety requirements (https://echa.europa.eu/).

It has been argued that the most effective way of preventing pollution and complying with related legislation is that of designing chemicals that are inherently safer or benign by design (Anastas, 1994). In 1998, Anastas and Warner (1998) put forward the twelve principles of Green Chemistry, a concept that focuses on the environmental impact of chemistry. In addition to addressing safety by design, these principles also address aspects associated with environmental sustainability such as waste prevention, atom economy, energy efficiency, degradation and use of renewable resources. Anastas and Zimmerman (2018) have stated that the UN Sustainable Development Goals can only be advanced by chemistry *“designed to be sustainable such as that outlined and practiced through green chemistry”*.

Some of the principles of Green Chemistry have also been introduced into the nanotechnology field, for example within the Safe-by-Design (SbD) concept as outlined by the OECD (OECD, 2020). Safe-by-Design (OECD, 2020) aims to identify the risks and uncertainties concerning human health and the environment at an early phase of the innovation process to minimize them before the regulatory registration/approval procedure is initiated. In general, SbD involves (iterative) stage-gate safety assessment based on the Cooper Stage Gate innovative model (Cooper, 1990) that divides the innovation process into a predefined set of stages, each including specific activities. The progression from one stage to the next is regulated by a gate, where the project is evaluated according to a set of adaptable safety criteria, leading to a decision on whether to continue the project or not. Case studies for applying the SbD approach have been performed by the nanotechnology industry (Sánchez Jiménez et al., 2020).

The new approach advocated in the EU Chemicals Strategy for Sustainability for ensuring sustainability as well as safety, i.e., Safe- and Sustainable-by-Design (SSbD), goes beyond just adding sustainability considerations on top of an established SbD process. It explores the interlinkage between safety and sustainability from a systems perspective and requires that a set of SSbD criteria are incorporated in the process. In order to achieve the paradigm shift towards a circular economy envisaged in the Green Deal, true integration of safety and sustainability throughout the chemical product development process is needed. It involves, for instance, the substitution of substances/materials by safer and/or more sustainable items as well as a complete re-design of the entire innovation process. Gottardo et al. (2021) have described the significance of SSbD for nanotechnology and Smart NMs.

The development of clear, internationally agreed and quantitative (i.e. measurable) sustainability criteria is a logical and necessary step forward. Different criteria may be needed for different kinds of products, and the challenge will be to define not only the criteria but also the thresholds for deciding when a substance or product is deemed sustainable according to each criterion. The development of such criteria by the European Commission has just started (Amodio et al., 2021; European Environmental Agency, 2021; World Business Council for Sustainable Development, 2018).

The EC is committed to developing a framework and criteria for SSbD by the end of 2022 (European Commission, 2020b, 2020c). For this purpose, the EC prepared a mapping study to collect insights into existing frameworks in order to develop SSbD criteria for chemicals, materials and products (Amodio et al., 2021), followed up by a Stakeholder Survey launched in June 2021. The EC aims to publish outcomes of this work in 2022, expected to contain more explicit options for a transition to SSbD. Furthermore, new actions proposed under the Horizon Europe Programme 2021–2022 aim at the further development, testing and validation of the SSbD concept. Regarding a possible rule-based approach towards more sustainable products, it is worthwhile to point out the EC's Product Environmental Footprint Pilots (https://ec.europa.eu/environment/eussd/smgp/ef_pilots.htm) which included as objectives the testing of the process for developing product- and sector-specific rules (product environmental footprint category rules) and of different approaches to verify such rules in several pilot studies.

Furthermore, a call for a dedicated coordination and support action has been launched (Horizon Europe, 2021) to create an EU-led international community on materials that are safe and sustainable by design to support embedding sustainability criteria over the life cycle of products and processes. This will contribute to the implementation of the Chemicals Strategy for Sustainability and thus support the transition in which safety and sustainability of chemicals become essential market entry conditions for any product. The need for establishing a broad international community on SSbD to support the development and implementation of sustainability criteria was emphasised at the workshop. An operational, not only conceptual, framework for SSbD would promote a closer, creative co-operation between specialists and multidisciplinary teams and developers with different mind sets. In addition to a common framework, sector-, material- and use-specific criteria to account for specific scenarios should also be included. After the workshop, also an International Network Initiative on Safe and Sustainable Nanotechnologies was proposed (Falk, 2021).

The international community on nanosafety has already taken initiatives in that direction. For example, NanoReg2 developed a general SbD framework that also accommodates sustainability considerations (environmental impact) by using Life Cycle Assessment (LCA) and socioeconomic assessments. Additionally, other projects such as SUN (SUN: Sustainable Nanotechnologies: http://www.sun-fp7.eu/) and SUNSHINE (SUNSHINE: https://www.h2020sunshine.eu/) focus on developing criteria, guiding principles and tools to assess sustainability of nanotechnology-based products (SUN, 2017), including those based on Smart NMs. Possibly, the network on nanosafety could be expanded to also address sustainability by including more actors from that field.

In addition to defining a framework and related criteria, it would be beneficial to integrate SSbD in educational programmes at academic level to train a new generation of material and product developers on how to apply SSbD in industrial settings. At the same time, engagement in implementing a SSbD approach in industrial settings could be promoted by incentives such as a prolonged patent duration or, if authorisation/approval is required, a fast track evaluation of chemicals developed and produced according to an SSbD approach.

**Fig. 1:**
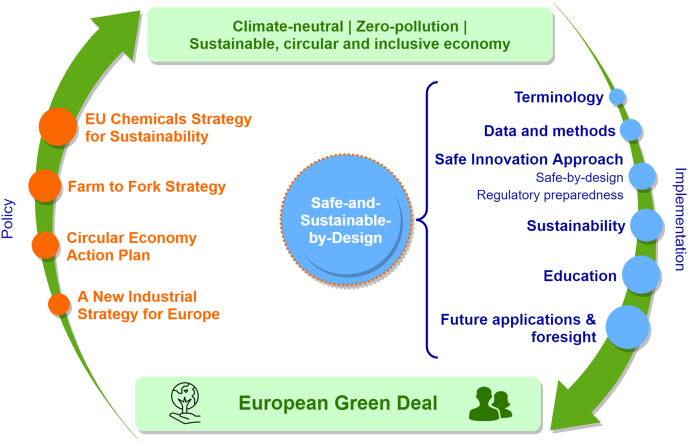
Policy initiatives and action plans to be considered in the development of Safe-and-Sustainable-by-Design (SSbD) materials (left side), in alignment with the proposed elements for a SSbD approach (right side).

Hence, development of methods for ensuring that future chemicals, including Smart NMs, are safe and sustainable will be an important contribution to the stepwise translation of the policy visions of the European Green Deal into the reality of a sustainable, circular and inclusive economy. This is illustrated in Fig. 1: on the left side, policy initiatives and action plans are mentioned which would be addressed by implementing the SSbD concept. The right side of Fig. 1 describes the elements of a SSbD approach as discussed in this paper.

## 3 The Smart Nanomaterials case

### 3.1 The need for an agreed terminology

Agreed terminology fosters mutual understanding between scientists, industry and regulators within the same field, and it is necessary for defining the applicability domain of legislation. Gottardo et al. (2021) gave examples of Smart NMs and concluded that there is yet no harmonised description or definition of what a ‘smart nanomaterial’ is, noting that such materials are also known as stimuli-responsive, multifunctional or active nanomaterials.

*Smart Nanomaterial* is a combined term: the first half, ‘smart’, refers to these materials having a stimulus-triggered function. The second half, ‘nanomaterial’, is defined by the European Commission in an EU regulatory context (EC NM Definition) (European Commission, 2011), as well as by ISO (International Organization for Standardization). The EC NM Definition has been introduced as such or in a modified form in different pieces of legislation (European Parliament and Council, 2006; European Parliament and Council, 2012; European Parliament and Council, 2017; European Commission, 2018). Some EU legislation (Rasmussen et al., 2019a; Rauscher et al., 2017) defined nanomaterial before the EC NM Definition was adopted, and thus the term is not yet univocally defined across EU legislation. Currently, the EC NM definition is being reviewed and the outcome is expected to form the basis for harmonising the term across EU legislation, also contributing to Regulatory Preparedness. The ongoing work to define the term “*nanomaterial”* is carried over to derived terms, such as *Smart Nanomaterial*. It should be noted that nanotechnology-based multi-functional or active materials include nanostructured materials that are not necessarily covered by the EC NM Definition (Rauscher et al., 2019), as discussed in a recent study (European Chemicals Agency, 2019a).

Smart NMs are not explicitly mentioned in EU legislation, and the scientific literature gives different understandings of what Smart NMs are and their naming (Gottardo et al., 2021). Thus, a widely accepted and precise description (not necessarily a formal definition) of the term Smart NM would be useful by establishing a level playing field for both regulators and innovators, also in a regulatory context.

It would be helpful to first reach a consensus on the conceptual boundaries of the term *Smart NM* and, once agreed, illustrating them with examples of materials in order to establish a commonly agreed description of Smart NMs. A study by ECHA contains such examples for the case of defining the different “generations of nanotechnology applications” (European Chemicals Agency, 2019a). The same study also notes that it is sometimes unclear which of the REACH terms *substance*, *mixture* or *article* is the best fit for some Smart NMs and other advanced materials. Moreover, boundaries between different materials are not always clear. In any case, the starting point for developing terminology could be to base it on the functions exerted by Smart NMs. A response to one or more external stimuli, foreseen in the design of the material, could be a general feature that characterises Smart NMs (Gottardo et al., 2021).

Moreover, Smart NMs can be seen as a subgroup of the more generic *advanced materials,* which are explicitly referred to in the EU Chemicals Strategy for Sustainability and the EU New Industrial Strategy. An attempt to frame the term *advanced materials* has been made by the German Environment Agency (Umweltbundesamt, 2021; Giese et al., 2020) and also the ISO Committee ISO/TC 229 (Nanotechnologies) is discussing how to define *advanced materials*.

Another important step towards the common understanding and sharing of information on Smart NMs would be to develop a harmonised ontology for naming these materials and to represent other knowledge related to their distinctive aspects, e.g. the response to external stimuli and other the specific functions exerted by Smart NMs. This may be challenging, as Smart NMs and advanced materials in general are each a very heterogeneous group.

Hence, there is a need for a common understanding of the term *Smart Nanomaterials* for regulatory purposes, and a common understanding of the term *advanced materials* should also be developed. However, progress in the assessment of safety and sustainability of these materials must not be blocked by the lack of harmonised terminology.

### 3.2 Safety, safe-by-design and sustainability

The intended shift from SbD to SSbD is described above in section 2. When aiming to integrate the SbD in a new SSbD concept, it is useful to analyse how and when safety, sustainability and design aspects are currently considered in the material development process, using Smart NMs as an illustrative example. This section describes these different components.

#### 3.2.1 Safety of Smart Nanomaterials

As with all materials, the inherent properties of Smart NMs affect their safety, and as with other NMs (European Commission, 2018), physical-chemical properties, such as composition, particle size, surface properties (e.g. charge) and particle shape, influence the safety profile of Smart NMs. In addition, their stimuli responsiveness may also have an effect. It should be noted that there is no single property which determines the safety of all Smart NMs. There is not yet a common view among scientists and regulators on which additional parameters and properties it would be relevant to consider when assessing the safety of Smart NMs. The considerations expressed at the workshop, as well as additional considerations by the authors, are summarised below:

• The externally applied stimuli should be considered in the safety assessment of Smart NMs as they may trigger safety-relevant changes in the properties of Smart NM. A detailed description of such stimuli and the mechanism, timing and/or location for the triggered response would be essential to evaluate the safety of Smart NMs.
• The dynamic behaviour of Smart NMs along their life cycle implies that their physical-chemical properties and structure change in time and space due to external stimuli. This will likely result in life-cycle stage dependent safety profiles, by affecting for example toxicokinetics, environmental fate, bioavailability and toxicity. The long-term fate and behaviour of Smart NMs and any implications for exposure and the hazard profile also needs to be clarified. The degradation and aging of Smart NMs, which may also include disassembling, may also affect their designed responsiveness and cause e.g. a response to unintended stimuli. These aspects should be investigated using appropriate tools/tests.
• The designed smart response, or a different and undesired response, may be triggered unexpectedly in the environment or within living organisms (e.g. in various organs). It must therefore be ensured that unintended interactions and consequent adverse effects do not occur.
• The test conditions need attention, as the dynamic behaviour may depend on the test medium and experimental setup. It is therefore important to understand, possibly through new research, how the materials perform both under laboratory conditions, and in complex real-life environments.
• Existing tools, including methods, models and assessment approaches, should be evaluated by experts in the light of the dynamic behaviour of Smart NMs. The aim would be to identify to what extent and which kind of adaptations of the tools are needed, or if new ones should be developed, to specifically investigate the mechanisms by which Smart NMs exert their functions and possible toxic effects. This could be based on development of knowledge of the physiological and (eco)toxicological behaviour of Smart NMs.
• Developing new in situ imaging techniques for analysing the interaction between a bio-responsive system and the target tissue(s) is fundamental for Smart NMs with an intended function to be exerted within the human body.
• The applicability of OECD test guidelines to first generation, ‘conventional’ NMs has been reviewed (OECD, 2009), (Rasmussen et al., 2019b). Currently the OECD is updating some of them and developing new ones as needed (Rasmussen et al., 2019b); their applicability to Smart NMs may need to be evaluated and confirmed. A similar review may be needed for ISO standards for nanomaterials. To ensure that test methods, tools and guidance will be available for Smart NMs, an action similar to the “Malta Initiative” (Malta Initiative, 2017) could be considered, possibly supported by information generated by the EU NanoSafety Cluster. (https://www.nanosafetycluster.eu)
• The research needs for filling any gaps in knowledge and methodology should be identified.
• Regarding the multicomponent nature of Smart NMs, research and appropriately designed testing will be important to understand to what extent information from the individual components can be used for safety assessment, and whether mixture and combination effects can be expected. The possibility of such effects is a complicating factor for the testing and risk assessment of these materials, as e.g. the standard additive effect-calculation approach does not capture synergistic/antagonistic effects.
• In the future, grouping and read-across need to be considered, but first a solid body of (FAIR) data to support this approach needs to be produced.

The dynamic nature of Smart NMs may influence risk assessment and governance (Subramanian et al., 2010; Howard, 2011; Suominen et al., 2016). Possible safety concerns over Smart NMs are outlined in a recent review (Gottardo et al., 2021). For hazard and risk assessment of Smart NMs, it is crucial to consider their whole life cycle and possible changes in properties as described above. It is also important to identify and define the context of safety, as the range of accepted risk/uncertainty can depend on the sector of application. The risk assessment of Smart NMs poses new challenges due to their complexity and dynamic behaviour, which could increase their risks and, at the same time, require a higher level of knowledge to assess those risks (International Risk Governance Council (IRGC), 2007). Issues associated with the possible changes in inherent properties over time, combined with concerns over consumer exposure to such materials, have been pointed out in a survey (Sadowski and Guston, 2016).

The challenges in assessing the risk of Smart NMs can be illustrated by an example of human health risk assessment of Smart NM applications in a cosmetic product. The cosmetic product contains nanospheres loaded with multiple active ingredients that can be delivered, for instance, upon changes in pH or after reactions promoted by specific enzymes present in the skin, as developed for example in the PeptiCaps project (https://cordis.europa.eu/project/id/686141). In this system, the encapsulation protects the active ingredient from degradation, the capsules then degrade for example with the natural pH change across the epidermis, releasing the active ingredients at the target site with a significant increase of the efficacy and bioavailability. In addition to the challenges already existing for assessing nanomaterials there are other, specific challenges linked to Smart NMs.

• If the Smart NM consists of different components, the assessment should address the combination of all components together as one nano-sized entity. However, data on the ensemble of all components combined into a nano-sized entity are often scarce.
• Detailed characterization of the Smart NMs, including their activity/function is essential.
• In case of systemic exposure, toxicokinetic data are needed and should indicate the activity and the potential site(s) of activity/function.
• As indicated previously, Smart NMs are more complex to assess as, for example, the unintended consequences of the Smart NM performing its activity/function in unforeseen locations in the body (for example upon release of the encapsulated substance triggered by an unexpected stimulus) need to be considered as well.

The guidance on safety assessment of nanomaterials in cosmetics (SCCS, 2019) addresses nano-carriers and nano-encapsulated materials and incorporates already some of the above-mentioned considerations.

It should also be noted that in the EU, the use of test animals for generating new toxicological information for assessing the safety of cosmetic products and ingredients is banned. However, most existing alternative methods have not (yet) been validated for NMs, and new nano-specific methods are not yet available or validated (Rasmussen et al., 2019b). These issues also need to be resolved for Smart NMs. Recent EU projects, such as PATROLS (https://www.patrols-h2020.eu/), have already made important progress in the development of alternative methods for safety testing of NMs. To ensure continuity and to avoid duplication of work, these earlier results are envisaged to be taken up by other EU projects, for example RiskGONE, SUNSHINE and HARMLESS (currently running), as well as three new projects which have been launched in 2021 (RISK-HUNT3R, PrecisionTox, ONTOX). These projects should help to mature the previously achieved results towards validation of methods via collaboration with networks of laboratories, such as EU NETVAL (European Union Network of Laboratories for the Validation of Alternative Methods).

Overall, the risk assessment of Smart NMs poses new challenges due to their complexity and dynamic behaviour which, compared to conventional chemicals or NMs, could increase their risks and at the same time require a higher level of knowledge to assess those risks (International Risk Governance Council (IRGC), 2007; Sadowski and Guston, 2016).

Activities to reach a higher level of knowledge have already started in various fora, and furthermore, a closer dialogue between innovators and regulators would certainly help to reduce the uncertainty in assessing the risks of Smart NMs as well as promote regulatory preparedness. The EC has established several working groups supporting the implementation of the Chemicals Strategy for Sustainability ((European Commission, 2020b) which, among other things, address the safety of advanced materials. The EC also supports several research actions targeting advanced materials. These are the Horizon 2020 projects ASINA, SABYNA, SABYDOMA and SBD4NANO, launched in 2019 within the topic “Safe by design, from science to regulation: metrics and main sectors”. In addition, within the topic dedicated to “Safe by design, from science to regulation: multi-component nanomaterials”, launched in 2020, the EC funds the projects SUNSHINE, DIAGONAL and HARMLESS. Direct involvement of both researchers and regulators in such projects fosters the dialogue between them. In the current work programme, Horizon Europe, the EC already integrated the aspect of sustainability in several new topics: “Safe- and sustainable-by-design polymeric materials”; “Safe- and sustainable-by-design metallic coatings and engineered surfaces”; “Safe- and sustainable-by-design organic and hybrid coatings”. Projects funded under these topics will aim at promoting the availability, affordability, sustainability and security of the supply of essential chemicals and materials in Europe. The EC also supports establishing an EU-led international community on safe- and sustainable-by-design materials to support embedding sustainability criteria throughout the life cycle of products and processes. The outcomes of these projects and initiatives can be expected to be relevant for Smart NMs as well.

Advanced materials (including smart NMs) are now included in the mandate of the OECD Working Party on Manufactured Nanomaterials (WPMN), which has established the new Steering Group Advanced Materials (SG AdMa). The SG AdMa aims to clarify whether current regulatory approaches are fit for AdMa, identify gaps for assessing their safety (and sustainability) and identify the extent to which certain AdMa may pose a concern. One of the goals is to recommend options of actions for decision makers, e.g. on needs for risk management, research, assessment methods and information requirements.

Moreover, the European Observatory for Nanomaterials (EUON), hosted by ECHA, is conducting up to three studies annually to address knowledge gaps for NMs that are of interest to the general public and the research community. Among the upcoming studies selected for 2022, one of the topics is devoted to graphene and graphene oxide based NMs. Graphene and relevant NMs are considered as promising candidates for Smart (stimuli-driven) NMs as nanocarriers for targeted delivery, for example in the agrochemical sector.

#### 3.2.2 Safe-by-Design for Smart Nanomaterials

According to OECD, the SbD concept (OECD, 2020) (Box 1) refers to identifying the risks and uncertainties concerning human health and the environment at an early phase of the innovation process to enable minimisation of uncertainties, potential hazard(s) and/or unintended exposure. For Smart NMs the SbD stage gate criteria may be associated with e.g. material functionalities, uses and exposure. It is important to note that at each gate, regulatory requirements are firstly considered, and hence the requirements applicable to a specific project must be understood. Several implementation tools have been developed (ProSafe, 2021; Jantunen et al., 2017, 2018b; Gottardo et al., 2017; Varsou et al., 2019). These concepts and tools are in principle also applicable to Smart NMs, and hence industry has tools available for addressing the safety of new (smart) nanomaterials at an early stage. At the workshop developers expressed familiarity with the SbD thinking; they reported to often apply the same (or similar) ideas as those behind SbD without explicitly referring to it.

For SbD, data enabling hazard assessment at the early stages of the innovation process are often unavailable. To address this, a combination of alternative testing approaches and grouping and read-across (Giusti et al., 2019; Mech et al., 2019), which is currently being developed for nanomaterials (Stone et al., 2020), could ensure the availability of sufficient data to perform a hazard analysis early on. Some alternative methods for the early stages of SbD are already available, such as *in vitro* screening methods that predict hazards. The EC has thus proposed to expand SbD, suggesting the wider approach of Responsible Research & Innovation (RRI) (European Commission, 2020d), (van den Hoven et al., 2013). RRI is a science policy (Owen et al., 2019) that can help to improve stakeholder participation and consider their perceptions when designing materials, including Smart NMs, assessing risks and making trade-offs. SbD may help to translate existing knowledge, acting as a bridge between environmental risk assessment and RRI.

Some challenges may be encountered when introducing SbD for Smart NMs. These include identifying the possible needs for adapting risk assessment methodologies and associated tools, and implementing these changes, as relevant.

The main driving factor for developers of Smart NMs is the performance of the material, i.e. how well it executes the desired design-inherent function. Standards, legislation and SbD are often of secondary concern in the (early) development phase. In practice, the SbD concept (OECD, 2020) mostly seems to be applied only just before the prototype level, i.e. when the product approaches Technology Readiness Level (EARTO, 2014) (TRL) 7, which is too late in the R&D process for SbD to be fully integrated in product development. Examples for exceptions are certain applications of plant protection products, including those utilising Smart NMs, where safety is considered already during the design phase.

#### 3.2.3 Sustainability of Smart Nanomaterials

The term sustainability is a widely used term but lacks a clear definition (Morelli, 2011). It is usually described as a three-legged approach simultaneously benefitting the economy, society and environment (Morelli, 2011) but the overall meaning of the term or the meaning of each component may vary field by field. Sustainability assessment is one of the most complex appraisal procedures and is usually conducted for supporting decision-making and policy in a broad environmental, economic and social context, thus transcending a purely technical/scientific evaluation (Sala et al., 2015).

It is a priority to have a clear, commonly agreed description of what sustainability and its assessment exactly mean for advanced materials, including Smart NMs. Sustainability is more complex to assess than safety because it also requires characterisation of a variety of aspects associated with the environmental, economic and societal dimensions and, moreover, these three pillars are inter-related. As with any technology, ensuring sustainable innovation would require that any new Smart NM or enabled product shows intergenerational benefits for human life, social systems and circular economies. At the same time, there should be no or minimised adverse effects along the life cycle in a variety of areas including, for instance, human health and the environment, natural resources consumption, energy demand, waste production and global warming.

The assessment of environmental sustainability of a material or product has been operationally defined by Morelli (Morelli, 2011) as *“meeting the resource and service needs of current and future generations without compromising the health of the ecosystems that provide them”* and is often associated with general aspects such as minimal consumption of energy and resources during manufacturing or use, possible re-use of the whole material or of its components, and recycling of raw materials. When considering sustainability, the potential trade-offs need to be investigated. For instance, the durability of a material is usually perceived as a property positively contributing to sustainability as it may allow extending the lifetime of a product containing the material, but it may become a negative aspect upon environmental release as it may lead to persistence. Likewise, the smart behaviour may lead to a reduction in material use, energy consumption and greenhouse emissions compared to non-smart solutions, for example in the targeted delivery of a pesticide or fertilizer to crops. However, the multicomponent structure of some Smart NMs may make the manufacturing process more energy intensive or hamper the recovery and recyclability of materials with vulnerable supply chains (e.g. elements such as palladium or ruthenium). Kümmerer et al. (2020) have argued that reducing the complexity in the design of molecules and product components is a necessary condition to integrate chemistry into a circular economy. This is especially relevant for Smart NMs, and other (advanced) materials that are characterised by a multicomponent structure.

Therefore, it seems necessary to identify and agree on a list of elements to consider when characterising the environmental sustainability of Smart NMs and enabled products. It may also be useful to develop specific criteria that define for each element a minimum or acceptable level of sustainability that should be achieved in the design of a Smart NM or enabled product.

Tools to evaluate the environmental sustainability of a product already exist. Life Cycle Assessment (LCA) is a well-known comprehensive approach that quantifies the environmental impacts of a product or system over its complete life cycle (European Commission-JRC, 2010). ISO defines life cycle as “consecutive and interlinked stages of a product system, from raw material acquisition or generation from natural resources to final disposal” and LCA as “compilation and evaluation of the inputs, outputs and the potential environmental impacts of a product system throughout its life cycle” (International Organization for Standardization, 2006). Examples of considered impact categories are global warming, eutrophication, depletion of resources, (eco)toxicity, air pollution, use of renewable and non-renewable energy, waste production, materials for recycling and energy production. LCA is often used to compare options to inform decision-makers regarding product or process design or redesign (Hauschild et al., 2020), for instance, a product containing a nanomaterial compared with a conventional alternative. LCA demands substantial data input, and such data may often be scarce. ISO requires a critical review of the quality of the LCA study by a third party before bringing it to the public.

LCA is considered a suitable tool to assess the environmental sustainability of nanomaterials and nano-enabled products (Klöpffer et al., 2007) but there is a need for more data to build a Life Cycle Inventory that takes all nano-related functionalities into account. Existing models so far seem to fail to characterise correctly releases and impact factors of nanomaterials (Salieri et al., 2018), (Beloin-Saint-Pierre et al., 2018). Applying LCA principles would be suitable also for Smart NMs, but the data needs could be even bigger than for nanomaterials and the model development may become more complicated. Gate-to-gate data for the production of a material needs to be collected in the Life Cycle Inventory, which is already challenging for nanomaterials and will be even more so for Smart NMs. The study design also has to take into account stimuli-responsive functionalities that change during use. To this end, consensus should be found on the most relevant data input for Smart NMs. In addition, existing models to characterise the release of nanoparticles into the environment may not be adequate for Smart NMs. These methodological and data gaps may hamper the applicability of LCA to Smart NMs at early stages of design, when the available knowledge base on the material to develop is still small. Falinski et al. (2018) have suggested a less data intensive approach to assess nanomaterial sustainability, which is applicable at the design phase. These authors expanded the Ashby material selection charts to include metrics that characterise not only function but also hazard (e.g. zebrafish data), environmental impacts (e.g. cumulative energy demand along the life cycle) and economic performance (e.g. price). The approach is flexible and can consider a large number of metrics simultaneously, if needed.

### 3.3 Data and data sharing

Safety assessment, SbD approaches as well as the evaluation of sustainability are based on data. An overview of available data is fundamental for identifying gaps and to promote the use of the available data to the maximum. When developing a new material, existing suitable data from other materials could be useful for predicting hazards via grouping and read-across. This approach may be considered for the safety assessment of Smart NMs as well (Stone et al., 2020). Appropriate data should be “FAIR”: findable, accessible, interoperable and reusable (Wilkinson et al., 2016). To achieve this, the data need to be shared in its raw format along with sufficient metadata and made available in an environment that will continue to exist and be findable. Data sharing relies on harmonised testing methods and data formats. An overview of the state-of-the-art for FAIR nanosafety data has been provided recently (Jeliazkova et al., 2021). Data quality, though not explicitly covered by the FAIR principle, is important for comparability, interoperability and re-usability (Comandella et al., 2020) and, for Smart NMs in particular, the databases should be able to collect complex data.

The data needed are often not shared in a timely or effective manner, e.g. because of issues related to intellectual property rights. Restrictions on data sharing often are an issue within research consortia, and clear rules are needed for academia to share the data at the right time to be useful to the industry and regulators, while still allowing the data owner to exploit the publication potential and other intellectual property rights. There are ongoing efforts and incentives to facilitate mutual data sharing, and the necessary infrastructure is evolving (Jeliazkova et al., 2014; Haase and Klaessig, 2018) to enable adaptation to the needs of different communities.

Recently, an EU Regulation (European Parliament and Council, 2019) entered into force that aims to improve the transparency of risk assessment in the food chain as well as the reliability, objectivity and independence of the studies used by the European Food Safety Authority (EFSA). It foresees public access to the data used by the Authority and ensures that EFSA is informed of all studies commissioned by industry and carried out by laboratories.

All these activities aim at pooling European data in key sectors, with common and interoperable data spaces to facilitate the data sharing among interested stakeholders. To promote and ensure data sharing, the coordination of FAIR e-infrastructures (knowledge bases, databases), including raw data and metadata, should be done at an overarching level that in Europe could involve the European Commission or an EU agency.

## 4 Regulatory preparedness

Regulatory Preparedness (RP) refers to the capacity of regulators and policymakers to anticipate the regulatory challenges posed by emerging technologies such as nanotechnology, particularly human and environmental safety challenges (OECD, 2020). This knowledge would enable adaptable legislation to be developed that can keep up with the pace of knowledge generation and innovation. Given the complex nature of Smart NMs, dealing with RP is particularly important for these materials. Hence, an analysis is needed to understand the preparedness and appropriateness of the EU regulatory framework and potential gaps in order to address the safety and sustainability of Smart NMs. Such an analysis, performed for nanomaterials (European Commission, 2012), has already led to amendments of EU legislation. As discussed in section 3.1 on terminology, there is a need for a common understanding of the term “*smart nanomaterial*” also in legislation. In order to assess whether and how the EU regulation addresses Smart NMs, a widely agreed unambiguous description or definition of these materials is needed. Currently, Smart NMs are not referred to in EU legislation nor are they explicitly defined for regulatory purposes (Gottardo et al., 2021).

An analysis is needed to identify relevant adaptations of existing legislation. When revising legislation to keep it up to date, legislators should strive for provisions, which are innovation-friendly and enable proactive adaptation to newly identified and emerging issues. As the analysis and possible adaptation of the regulatory frameworks may take some time, strategies for interim solutions to ensure the safety and sustainability of Smart NMs should be considered. They could include increasing scientific and regulatory expertise regarding Smart NMs, the design of learning tools, which might prove specifically useful for small and medium-sized enterprises, and the facilitation of data sharing while protecting data ownership. A next step could be the extension of academic curricula (of chemists, physicists, material scientists, engineers etc.) to include courses on toxicology, Safe-by-Design and Sustainable-by-Design.

RP of EU legislation and regulators for addressing safety issues has been discussed for innovation in nanotechnology (Jantunen et al., 2018a), including measures to improve the level of preparedness, if relevant. Given their ‘smart’ properties, this discussion is even more relevant for Smart NMs.

The current regulatory data requirements for nanomaterials in the EU have been developed to address first-generation nanomaterials, and hence their adequacy for subsequent NM generations, including Smart NMs, needs to be confirmed (European Chemicals Agency, 2019a). Referring to an earlier analysis of how REACH, the Cosmetic Products Regulation (CPR) and food legislation address Smart NMs (Gottardo et al., 2021) and considering the current state of knowledge of safety assessment, the workshop experts concluded that the current regulatory frameworks seem to be generally adequate and suitable for addressing the safety of Smart NMs. However, it is also recognised that additional characterisation parameters are needed to capture the dynamic dimension of second- and third-generation nanomaterials (European Chemicals Agency, 2019a; Gottardo et al., 2021). Such parameters should reflect the type of stimulus, the intended function and the type of change that the nanomaterial undergoes, including changes in function along the life cycle of the material or product, which confirms an earlier analysis (Gottardo et al., 2021). For the data requirements, a move towards more flexible approaches to testing and assessment, in which requirements are adaptable to the ‘smart’ properties, functions, uses etc. of Smart NMs, should be reflected upon. The lessons learned from adapting REACH and other legislation to specifically address nanomaterials would be a good basis to build on. Also best practices from other areas where nanomaterials are regulated, such as those for medicinal products and medical devices, should be collected. It would be relevant to identify common data requirements, assessment approaches and tools among the several legislative areas for confirming/establishing any additional requirements.

While legal provisions exist in the EU to ensure that all products placed on the market are safe for human health and the environment, a similar comprehensive set of requirements to ensure that products are sustainable is currently missing. The EC has recently decided to develop an overarching policy framework for sustainable products making the Ecodesign Directive (European Parliament and Council, 2009), currently used for energy-related products, applicable to the broadest possible range of products, especially textiles, electronics, furniture and high impact intermediary products such as steel, cement and chemicals (European Commission, 2020c). The EC also intends to define sustainability principles addressing product durability, reusability, upgradability, reparability, energy and resource efficiency, carbon and environmental footprints as well as presence of hazardous chemicals (European Commission, 2020c).

Requirements for reaching the earlier UN sustainable development goals are built into EU legislation and the 2030 UN Agenda (United Nations, 2015) presents a new systems approach and updated goals that should be reflected in the EU chemicals legislation. With the Green Deal and associated policy initiatives, it is intended to make sustainability highly visible in the chemicals legislation by explicit requirements, beyond safety, and quantifiable criteria, as well as updating the guidance to strengthen the parts of assessment that could support sustainability.

For achieving RP (Jantunen et al., 2018a), the importance of events such as this workshop (European Commission Workshop, 2020) must be stressed, and further opportunities for discussion and information exchange among the relevant stakeholders would be highly beneficial. In addition, the establishment of expert networks as well as suitable tools for systematic horizon scanning would be useful. RP would benefit from information and data exchange that is as open as possible, and for which specific established trusted environments would be needed (Jantunen et al., 2018a).

For the legislative frameworks for chemicals, food, and cosmetics, guidance is available that specifically addresses data requirements and safety assessment of nanomaterials; the applicability of the available guidance to Smart NMs needs to be assessed. Specifically, the triggers for the functions of Smart NMs and changes along the life-cycle need to be considered, as well as how to evaluate the probability of a function failure and the risk derived from non-function. For the purposes of REACH, being able to decide whether a certain Smart NM should be considered as a substance, a mixture or an article (European Chemicals Agency, 2019a) would be essential for both industry and regulators to identify relevant legal requirements and assessment approaches. Hence, it would be useful if the “Guidance on requirements for substances in articles” (European Chemicals Agency, 2017), which outlines how to determine whether an object is an article under REACH, could be supplemented with examples of nanomaterials, including Smart NMs. Moreover, the REACH guidance on identifying nanoforms (European Chemicals Agency, 2019b) already addresses aspects related to the characterisation of nanoforms in line with the requirements of Annex VI of the REACH Regulation. Additional guidance maybe useful to describe and clarify specific aspects related to the characterisation of more complex multi-component materials such as Smart NMs. ECHA is currently updating the guidance on information requirements and chemical safety assessment to better address nanomaterials, and a similar update could be relevant for Smart NMs.

As discussed elsewhere (Gottardo et al., 2021), the European legislation covering the food and cosmetic sectors and associated guidance documents seems to be more prepared than other sectors to address functions and properties of smart materials. At the workshop, it was indicated that the health risk assessment of Smart NMs added to food and feed is challenging but feasible, provided that proper information is available, i.e. relevant characterisation data and adequately designed studies, and hazard data that relates to relevant human exposure. Additionally, methods to study migration from packaging into food require adaptation for Smart NMs. Furthermore, the EFSA principles for the environmental risk assessment of nanomaterials should be developed further to also address Smart NMs. Considering that for first-generation nanomaterials in food there is already an unmet need for reference materials, Smart NMs present an additional challenge. There is also a need for increased accredited laboratory capacity for the enforcement of the legislation.

For the purpose of CPR, the Scientific Committee on Consumer Safety (SCCS) has published guidelines for assessing nanomaterials in cosmetic products (SCCS, 2019); the applicability to Smart NMs of this guidance should be confirmed. Guidance would also have to be developed on how the current requirements and tools apply to Smart NMs and whether and how additional information should be considered. New developments in the field need to be monitored, in order to adequately and timely update guidelines for assessment and data requirements to ensure the provision of all relevant information. This would also enable an understanding of whether the assessment is appropriate, and to what extent the supporting evidence or data are relevant for the material. New (publicly funded) research programmes for promoting innovation could include an obligation to provide information also on (eco)toxicological aspects, which, in turn, would lead to fostering multidisciplinary projects and teams. A transparent and consistent dialogue and exchange of information among all stakeholders (e.g. developers, safety assessors, regulators, and society at large) should be ensured by establishing expert networks to discuss safety and sustainability considerations, including societal and ethical concerns. Concepts and initiatives mentioned above can be further supported by targeted education programmes to increase the expertise both for R&D and among regulators.

Table 2 provides an overview of the EU legislation referred to in the text, and includes also information on which European agency or scientific committee (if any) is involved in the legislation.

**Table 2:** Overview of EU legislation referred to in the text. None of these documents are explicitly addressing smart nanomaterials.

Application area and short reference	Full title of the document	Explicit reference to nanomaterials or nanotechnology?	Agency or scientific committee assisting the implementation^a^
**Nanomaterial definition** Commission Recommendation 2011/696/EU	Commission Recommendation of 18 October 2011 on the definition of nanomaterial (2011/696/EU)	yes	none
**Chemicals safety** **REACH** (Regulation, 1907/2006) and amendment (Regulation 2018/1882)	Regulation (EC) No 1907/2006 of the European Parliament and of the Council of 18 December 2006 concerning the Registration, Evaluation, Authorisation and restriction of Chemicals (REACH), establishing a European Chemicals Agency, amending Directive 1999/45/EC and repealing Council Regulation (EEC) No 793/93 and Commission Regulation (EC) No 1488/94 as well as Council Directive 76/769/EEC and Commission Directives 91/155/EEC, 93/67/EEC, 93/105/EC and 2000/21/EC *Amendment:* Commission Regulation (EU) 2018/1881 of 3 December 2018 amending Regulation (EC) No 1907/2006 of the European Parliament and of the Council on the Registration, Evaluation, Authorisation and Restriction of Chemicals (REACH) as regards Annexes I, III,VI, V	Yes (with the amendment through regulation 2018/1882)	ECHA
**Cosmetic Products** **Regulation** 1223/2009	Regulation (EC) No 1223/2009 of the European Parliament and of the Council of 30 November 2009 on cosmetic products	yes	SCCS (Scientific Committee on Consumer Safety
**Biocidal Products** **Regulation** 528/2012	Regulation (EU) No 528/2012 of the European Parliament and of the Council of 22 May 2012 concerning the making available on the market and use of biocidal products	yes	ECHA
**Novel food** **Regulation** 2015/2283	Regulation (EU) 2015/2283 of the European Parliament and of the Council of 25 November 2015 on novel foods, amending Regulation (EU) No 1169/2011 of the European Parliament and of the Council and repealing Regulation (EC) No 258/97 of the European Parliament and of the Council and Commission Regulation (EC) No 1852/2001	yes	EFSA
**Food Additives** **Regulation** 1333/2008	Regulation (EC) No 1333/2008 of the European Parliament and of the Council of 16 December 2008 on food additives	yes	EFSA
**Plastic Food Contact Materials** **Regulation** October 2011	Commission regulation (EU) No October 2011 of 14 January 2011 on plastic materials and articles intended to come into contact with food	yes	EFSA
**Active and Intelligent Food Contact Materials** **Regulation** 450/2009	Commission regulation (EC) No 450/2009 of 29 May 2009 on active and intelligent materials and articles intended to come into contact with food	yes	EFSA
**Provision of Food Information to Consumers** **Regulation** 1169/2011	Regulation (EU) No 1169/2011 of the European Parliament and of the Council of 25 October 2011 on the provision of food information to consumers, amending Regulations (EC) No 1924/2006 and (EC) No 1925/2006 of the European Parliament and of the Council, and repealing Commission Directive 87/250/EEC, Council Directive 90/496/EEC, Commission Directive 1999/10/EC, Directive 2000/13/EC of the European Parliament and of the Council, Commission Directives 2002/67/EC and 2008/5/EC and Commission Regulation (EC) No 608/2004	yes	EFSA
**Medical Devices** **Regulation** 2017/745	Regulation (EU) 2017/745 of the European Parliament and of the Council of 5 April 2017 on medical devices, amending Directive 2001/83/EC, Regulation (EC) No 178/2002 and Regulation (EC) No 1223/2009 and repealing Council Directives 90/385/EEC and 93/42/EEC	yes	JRC and EMA (European Medicines Agency)
**Waste Electrical and electronic equipment (WEEE)** **Directive** 2012/19/EU	Directive 2012/19/EU of the European Parliament and of the Council of 4 July 2012 on waste electrical and electronic equipment (WEEE)	yes	None
**Risk assessment in the food chain** **Regulation** 2019/1381	Regulation (EU) 2019/1381 of the European Parliament and of the Council of 20 June 2019 on the transparency and sustainability of the EU risk assessment in the food chain and amending Regulations (EC) No 178/2002, (EC) No 1829/2003, (EC) No 1831/2003, (EC) No 2065/2003, (EC) No 1935/2004, (EC) No 1331/2008, (EC) No 1107/2009, (EU) 2015/2283 and Directive 2001/18/EC	no	EFSA
**Ecodesign of products** **Directive** 2009/125/EC	Directive 2009/125/EC of the European Parliament and of the Council of 21 October 2009 establishing a framework for the setting of ecodesign requirements for energy-related products	no	None

a ECHA – European Chemicals Agency; EFSA – European Food Safety Authority; EMA – European Medicines Agency; JRC – Joint Research Centre, a Directorate-General of the European Commission; SCCS – Scientific Committee on Consumer Safety.

## 5 Conclusions and future actions

The workshop “*Safe and Sustainable Smart Nanomaterials*” introduced the concept of Safe- and Sustainable-by-Design (SSbD) of chemicals and products to stakeholders outside of the EC and discussed on how to obtain safe and sustainable Smart NMs as an example of the heterogeneous group of advanced materials.

The outcomes of the workshop have meanwhile served as input to related initiatives. The workshop was held just before the publication of the EU Chemicals Strategy for Sustainability and has contributed to the European Commission's recent actions on further developing the SSbD concept. In particular, a broad multidisciplinary group of experts on safety and sustainability needs to be consulted to reach consensus on SSbD principles and their implementation. In addition, a related robust educational framework at academic level should be established to ensure that material and product developers have the adequate knowledge and mind-set to apply SSbD in industrial settings.

Additionally, in March 2021 the EC hosted the 1st stakeholder workshop on SSbD criteria for chemicals, materials and products, in which three main activities towards the development and implementation of this concept were presented: i) establishing a framework for criteria development, ii) setting up an EU-wide network of experts and stakeholders, and iii) definition of sector/application specific criteria. Furthermore, in the Horizon Europe programme for 2021–2022, the EC has published a specific call (Horizon Europe, 2021) for creating an EU-led international community on SSbD materials to support embedding sustainability criteria throughout the life cycle of products and processes, as well as for testing and validation of the SSbD concept. This will contribute to the implementation of the Chemicals Strategy for Sustainability and thus support reaching the stage when not only safety but also sustainability will be considered essential and integral prerequisites for new products entering the EU market.

For the purpose of the workshop, Smart NMs and enabled products were chosen as a case study to understand whether there are research and regulatory needs that should be addressed as soon as possible to ensure safe and sustainable advanced materials. Based on the workshop discussions and further considerations reflected in this paper, a series of recommended actions proposed for scientists, regulators and innovators was identified:

i) Establishing harmonised terminology for Smart NMs: Smart NMs are a heterogeneous group of advanced materials. A harmonised description of the scope and boundaries of the term *Smart NMs*, as well as a systematic nomenclature and ontology of related terms should be developed. This could be achieved by creating an international network of scientists and regulators to work on this topic. On-going work within the OECD and ISO can provide input to the development of the terminology.
ii) Reaching a consensus on relevant parameters for assessing safety of Smart NMs: different experts and stakeholders should exchange data and information on their research activities to reach a consensus on the parameters to be considered when characterising Smart NMs and assessing their safety, with focus on parameters/properties linked to their dynamic (stimuli-responsive) nature.
iii) Defining information requirements and criteria necessary to assess the sustainability of Smart NMs and enabled products: in its most recent policy actions the EC has committed to the development of specific criteria and information requirements for assessing the sustainability of chemicals, products and processes in general. Considering that advanced materials, including Smart NMs, may soon become Key Enabling Technologies in crucial sectors, the sustainability criteria will need to take into account their specific structures, properties and functionalities.
iv) *Developing methodologies* for SSbD of chemicals, materials and products, including Smart NMs, e.g. through evaluating the degree of sustainability throughout the lifecycle. This would include the development of clear, internationally agreed and quantitative (i.e. measurable) sustainability criteria.
v) Assessing the appropriateness of available tools for risk assessment and LCA of Smart NMs: the available test guidelines and methods (including alternative methods), models and assessment methodologies need to be evaluated regarding their applicability to Smart NMs, also identifying to what extent adaptations or new tools may be needed. This could build on experiences gained previously under the OECD and NanoSafety Cluster umbrellas. Ongoing initiatives on generation of FAIR data for nanomaterials should be reinforced.
vi) Evaluating the adequacy of the current EU regulatory frameworks and existing associated guidance: a comprehensive review should be carried out in order to identify gaps and needs for adaptations to ensure that all properties/information relevant to Smart NMs are captured by relevant legislation and guidance documents. Such a review would also contribute to ensuring Regulatory Preparedness.

Implementing these actions would contribute to a stepwise transformation of the policy ambitions framed by the European Green Deal into reality, here exemplified by the case study of Smart NMs and enabled products.

## Disclaimer

The content expressed in this paper is solely the opinion of the authors and does not necessarily reflect the opinion of their institutions.

## Funding body information

This work was funded through the European Commission's 10.13039/501100000900Joint Research Centre, which is financed by the EU's Framework Programmes for Research and Innovation (Horizon 2020 and Horizon Europe).

## CRediT authorship contribution statement

**Agnieszka Mech:** Conceptualization, Writing – review & editing. **Stefania Gottardo:** Conceptualization, Writing – review & editing. **Valeria Amenta:** Writing – review & editing. **Alessia Amodio:** Writing – review & editing. **Susanne Belz:** Writing – review & editing. **Søren Bøwadt:** Writing – review & editing. **Jana Drbohlavová:** Writing – review & editing. **Lucian Farcal:** Writing – review & editing. **Paula Jantunen:** Writing – review & editing. **Aleksandra Małyska:** Writing – review & editing. **Kirsten Rasmussen:** Conceptualization, Writing – original draft preparation, review & editing. **Juan Riego Sintes:** Writing – review & editing. **Hubert Rauscher:** Conceptualization, Writing – original draft preparation, review & editing.

## Declaration of competing interest

The authors declare that they have no known competing financial interests or personal relationships that could have appeared to influence the work reported in this paper.

## Footnotes

2 In the literature the notations “Safe-by-Design”, “Safe(r)-by-Design” and other variations can be found for the same concept. The notation “safe-by-design” is used in this article.
